# Oxidative Stress Is a Double-Edged Sword for the Neonate

**DOI:** 10.3390/antiox15030400

**Published:** 2026-03-23

**Authors:** Ru-Jeng Teng

**Affiliations:** 1 Department of Pediatrics, Medical College of Wisconsin, Children Corporate Center, Suite C410, 999N 92nd Street, Milwaukee, WI 53226, USA; rteng@mcw.edu; 2 Children’s Research Institute, Medical College of Wisconsin, 8701 W Watertown Plank Rd., Wauwatosa, WI 53226, USA

Fetal oxygen tension is low, with an umbilical venous pO_2_ of 30–37 mm Hg in the third trimester, while arterial pO_2_ is around 20 mm Hg. Placental PaO_2_ can be as low as 18 mm Hg at 8–10 weeks of gestation. These values are relatively lower than those in ambient air, which is approximately 150 mm Hg. This “physiological hypoxia” is necessary for placental growth and tissue differentiation because it stabilizes hypoxia-inducible factors (HIF-1 and HIF-2) [1]. Acting as master regulators, HIFs promote vascular endothelial cell growth by upregulating glycolysis and inducing vascular endothelial growth factor (VEGF) expression [2]. This process is coordinated by reactive oxygen species (ROS) produced during “physiological hypoxia.” The right amount of ROS is vital for developmental senescence, which is essential for organ patterning during fetal development [3]. Therefore, an optimal level of ROS is indispensable during fetal growth.

The sudden increase in oxygen during the perinatal transition, with pO_2_ rising sharply from below 40 mm Hg to 150 mm Hg, can effectively turn off this HIF pathway. However, if neonates require supplemental oxygen for extended periods due to respiratory distress, oxidative stress (OS) can sequentially activate HIF1α and HIF2α. Activation of HIF1α during the initial phase is proinflammatory, while subsequent HIF2α activation supports angiogenesis and organ repair [4]. Persistent OS may cause organ damage by impairing mitochondrial function [5], triggering the unfolded protein response [6], autophagy [7], oxidation-induced senescence [8], and cell death pathways such as necrosis, necroptosis, pyroptosis, ferroptosis, and others [9], along with fibrosis [10]. The damage worsens if prematurity is involved, as premature neonates are born with surfactant deficiency and an underdeveloped antioxidative system. The oxygen supplementation and positive airway pressure required to maintain tissue oxygenation can further increase ROS production in premature infants, potentially overwhelming their immature antioxidant defenses.

Excessive ROS in sick premature neonates can damage cellular DNA, especially in rapidly dividing stem and progenitor cells. OS-induced DNA damage can lead to cell death or senescence, depriving immature organs of growth potential by depleting these progenitor cells [11]. At the same time, dead or dying cells release high-mobility group box 1 (HMGB1), which triggers innate inflammation [12]. This inflammation, mediated by cells containing inducible nitric oxide synthase and myeloperoxidase (MPO), releases MPO and NO, creating highly reactive oxidants such as hypochlorous acid (HOCl) and peroxynitrite (ONOO^−^) that damage immature organs [13]. Thus, inflammation amplifies OS. The interaction between OS and inflammation [14] creates a self-sustaining, destructive cycle in neonatal organs under respiratory support.

HMGB1 is a non-histone nuclear DNA-binding protein that is released from the cell upon injury. Once outside, HMGB1 acts as a danger signal, activating damage-associated molecular pattern (DAMP) signaling with proinflammatory effects [15]. Its receptors include the Receptor for Advanced Glycation End Products (RAGE) and Toll-like receptors (TLRs). When HMGB1 binds to these receptors, it activates NF-kB signaling, leading to macrophage and neutrophil activation. HMGB1 contains three sulfhydryl groups, and its effects depend on its redox state. Fully reduced HMGB1 acts as a chemoattractant, the disulfide form stimulates inflammatory cells, and the fully oxidized form is anti-inflammatory. Thus, HMGB1 is a key link between OS and inflammation.

Prematurity and intrauterine growth restriction predispose to OS and inflammation even before birth [14]. Paradoxically, the heightened OS upregulates the HIF pathway through ROS production. As a result, VEGF expression increases to maintain tissue oxygenation, but it can also lead to disorganized pathological angiogenesis (dysangiogenesis). This abnormal vessel growth is associated with several prematurity-related conditions, such as bronchopulmonary dysplasia (BPD) and retinopathy of prematurity (ROP), including the pulmonary hypertension seen in BPD [16]. Persistent activation of HIF signaling also promotes lung fibrosis via endothelial-to-mesenchymal transition [17]. Elevated HIF1α is also observed during the second ischemic phase of ROP. HIF1 can further boost insulin-like growth factor-1 (IGF-1) signaling by increasing IGF1 binding protein levels [18]. Interestingly, hyperoxia correlates with decreased IGF1, which strongly links to ROP development in meta-analyses [19]. These conflicting findings highlight the complexity of OS’s influence on HIF signaling in neonatal organs.

For a long time, OS has been considered the main cause of all prematurity-related complications. Over the past thirty years, many antioxidants have been studied, including N-acetylcysteine, vitamin C, vitamin E, superoxide dismutase, melatonin, selenium, and lactoferrin. Unfortunately, consistent clinical benefits have not been established. Although intramuscular vitamin A has been shown to reduce BPD [20], it is not an antioxidant itself. Several reasons may explain why broad-spectrum antioxidants do not work effectively: (1) basal OS is necessary for developmental senescence, which is vital for organ patterning and growth; (2) the timing of HIF1α and HIF2α changes is critical for organ recovery after injury; (3) OS plays complex roles across different organs simultaneously. Determining the precise level of OS that promotes premature organ growth while minimizing damage remains very challenging. Additionally, the interaction between OS and inflammation must be considered. A systems pharmacological approach targeting multiple signaling pathways might be the most effective strategy for managing OS-related neonatal disorders. Decades of research have led to a better understanding of OS in neonatal disorders. However, we are still far from finding the silver bullet. The animal models we have been using do not recapitulate what happens in premature neonates. Other animal models and new approaches will be needed to identify appropriate OS-oriented therapies.

In this Special Issue, entitled “Oxidative Stress in the Newborn”, we are excited to present five research articles and six reviews for our readers. In contribution #1, an ex vivo organotypic slice of a neonatal brain was challenged with oxygen–glucose deprivation and reperfusion (OGDR). Cell death, redox state, and proteomic profile of inflammatory mediators were studied in the corticostriatal and hippocampal regions. Melatonin was used as the therapeutic agent. Not surprisingly, OGDR increased cell death in both regions, but melatonin markedly attenuated it. Although melatonin increased the GSH/GSSG ratio in both regions, only the hippocampal region had a GSH/GSSG ratio below 1 under OGDR, indicating a regional difference in response to OGDR. In contrast, the OGDR-induced inflammatory response was more pronounced in the corticostriatal region, and melatonin also ameliorated it. In contribution #4, prenatal melatonin injection was shown to facilitate the postnatal lung maturation process in an antenatal inflammation-induced preterm birth. Together, these two contributions suggest that melatonin has both anti-inflammatory and antioxidative properties that can protect neonatal organs.

In contribution #2, Endesfelder and Bührer used a rat hyperoxia exposure model (80% O2) for 3 or 5 days to show that caffeine can restore alveolar development and limit the fibrosis. Although caffeine transiently enhances the fibrosis in room air-exposed fibrosis, in the end, there was no morphometric difference. Their study provided another mechanism by which caffeine attenuates BPD severity. In contribution #3, Shvetsova et al. demonstrated the maturational change in superoxide dismutase expression and activity, and their effect on the vasomotor activity of the systemic artery. Apparently, SOD and catalase activities are significantly higher in young rats (10–15 days) than in adult rats (3–4 months). The endothelial-independent vasomotor response in the systemic artery is more pronounced in young rats than in adult rats. The possible age-related difference in response should always be kept in mind when we treat patients.

In contribution #5, the milk composition was compared between vegan and omnivorous women. A significantly higher cortisol concentration and lower iron, vitamin B6, and antioxidant status were seen in the milk from vegan women than from omnivorous women. With the popularity of the vegan diet, health professionals need to be aware of the low iron and vitamin B6 content. Whether antioxidant depletion increases susceptibility to OS-induced organ damage warrants further exploration.

The cross-talk between the OS and inflammation has been clearly described in the literature. In contribution #6, Kaltsogianni et al. reiterated this relationship in several neonatal pulmonary disorders. With no obvious benefit from antioxidant therapy, they recommended judicious use of oxygen to avoid both hypoxia and hyperoxia, thereby reducing OS. The gut microbiome has attracted our attention over the past several years and has been linked to almost all diseases. In contribution #7, Morozan et al. suggested that maternal dysbiosis is associated with increased risk of perinatal asphyxia. The link is mainly through its association with maternal metabolic disorders, immune response, and impaired placental function, which might render an increased susceptibility to perinatal asphyxia in their offspring.

As inflammation results in direct organ damage and increased OS, corticosteroids were once almost routinely given to premature neonates due to the belief that their use could decrease the incidence of BPD. Later, a randomized controlled study showed a significant increase in neurodevelopmental deficit with early postnatal systemic steroid use, which led the American Academy of Pediatrics to recommend not prescribing systemic steroids to premature neonates [21]. Further studies suggested that the timing of steroid treatment might be critical for this association. In contribution #8, Galletta et al. carefully reviewed the topic and offered suggestions for addressing the issue.

In contribution #9, Teng et al. developed a new hypothetical destructive cycle based on their previous studies to explain the development and progression of BPD. The cycle is initiated by OS from supplemental oxygen treatment, followed by HMGB1-mediated DAMP signaling and the unfolded protein response, and then subsequent OS amplification, forming a self-perpetuating cycle. In this contribution, they suggested a systems pharmaco-therapeutic approach to prevent or attenuate BPD.

With E-cigarettes gaining more popularity nowadays, their impact on fetuses cannot be ignored. In contribution #10, Gambadauro et al. provided evidence that E-cigarettes are not as safe as users believe. Although caffeine has been shown to reduce BPD in premature infants through the Caffeine for Apnea of Prematurity (CAP) trial [22], its original hypothesis was that it reduces apnea of prematurity, thereby improving neurodevelopmental outcomes. Hypoxia, because of apnea and subsequent hyperoxia after recovery, is similar to the ischemic–reperfusion episode that will generate OS. Although the study did not show early-age neurodevelopmental benefits, its subsequent long-term follow-up did demonstrate efficacy. Because most enrollees were intubated early in the CAP trial, there was no way to demonstrate that caffeine reduced episodes of apnea of prematurity (AOP); it is impossible to show that AOP is associated with increased OS. This is described in contribution #11.

Unfortunately, we were unable to include all OS-related, prematurity-associated morbidities in our Special Issue due to time constraints. OS is believed to play a critical role in retinopathy of prematurity, acute kidney injury, necrotizing enterocolitis, periventricular–intraventricular hemorrhage, periventricular leukomalacia, and birth asphyxia. We hope researchers will offer their expertise and research to readers so that we can better understand pathophysiology and formulate the best ways to prevent or attenuate these morbidities.
